# An audit of the changes in thiamine levels during higher caloric nutritional rehabilitation of adolescent patients hospitalised with a restrictive eating disorder

**DOI:** 10.1186/s40337-020-00318-z

**Published:** 2020-09-01

**Authors:** Elizabeth Parker, Terri Maister, Anita Stefoska-Needham, Christine Wearne, Gail Anderson, Linette Gomes, Simon Clarke, Michael Kohn

**Affiliations:** 1 Department of Dietetics & Nutrition, Westmead Hospital, PO Box 533, Wentworthville, NSW 2145 Australia; 2 Sydney School of Health Sciences, Faculty of Medicine and Health, The University of Sydney, Sydney, 2006 NSW Australia; 3 SMART Foods Centre, School of Medicine, Faculty of Science, Medicine and Health, University of Wollongong, Wollongong, NSW 2522 Australia; 4 Department of Medical Psychology, Westmead Hospital, Westmead, NSW 2145 Australia; 5 Department of Adolescent & Young Adult Medicine, Westmead Hospital, Westmead, NSW 2145 Australia; 6 Sydney School of Medicine, Faculty of Medicine and Health, The University of Sydney, Sydney, NSW 2006 Australia; 7 Centre for Research into AdolescentS’ Health (CRASH), Adolescent & Young Adult Medicine, Westmead Hospital, Westmead, NSW 2145 Australia

**Keywords:** Thiamine, Vitamin B1, Eating disorder, Anorexia nervosa, Nutrition

## Abstract

**Background:**

Routine supplementation of thiamine in patients with restrictive eating disorders prior to initiation of nutritional rehabilitation, is an example of a clinical guideline based on expert opinion rather than evidence-based recommendations. This study investigates whether adolescents hospitalised with a restrictive eating disorder commenced on a higher caloric refeeding regimen, present with or develop thiamine deficiency during their admission.

**Methods:**

An eighteen month retrospective audit of 119 consecutive admissions for nutritional rehabilitation was conducted on patients admitted with an eating disorder in a large tertiary teaching hospital in Western Sydney. Data from paper-based and electronic medical records were collected. Baseline and weekly blood thiamine levels were documented, as well as patient demographic information including admission weight, age, length of stay, percentage median body mass index, weight change throughout admission and caloric prescription.

**Results:**

Sixty admissions met inclusion criteria, mean age 17.2 years (SD 1.2); 88% female; BMI 16.8 kg/m^2^ (SD 1.8) on admission. A linear mixed effects model identified that median thiamine levels increased by 9.2 nmol/L per week (*p* < 0.001). No patient developed thiamine deficiency during their admission, one patient was admitted with thiamine levels below the normal range at 62 nmol (normal range 67 – 200 nmol/L) which resolved by the second week of admission. In 15 out of 60 patients (25%), thiamine levels were observed to rise above the upper limit.

**Conclusions:**

Nutritional management of 60 malnourished adolescents hospitalised with an eating disorder was conducted safely with the provision of only 10 mg thiamine in a multivitamin daily, and no additional thiamine supplementation. The high caloric refeeding protocol, inclusive of a daily multivitamin, provided adequate thiamine to prevent thiamine deficiency. Further research should examine thiamine requirements in an exclusive severely malnourished population to assess the need for thiamine replacement in the most vulnerable group.

## Plain English Summary

This study looks at the change in thiamine (Vitamin B1) levels in 60 adolescent patients admitted to hospital with a restrictive eating disorder. Patients were provided with an oral meal plan with or without nasogastric tube feeding in hospital. Patients received a daily multivitamin providing 10 mg thiamine, however did not receive an additional thiamine supplement during their hospital admission. The results of this study found that no patients developed thiamine deficiency during their hospital admission, and that the average blood thiamine level increased each week. The results of this study suggest that this group of patients hospitalised with an eating disorder received adequate amounts of thiamine through an oral meal plan with or without nasogastric tube feeding, and a daily multivitamin, and did not require an additional thiamine supplement.

## Background

Nutritional management, incorporating refeeding and weight restoration (also termed nutritional rehabilitation), is a key pillar of the clinical care pathway of patients with anorexia nervosa (AN) and other restrictive eating disorders [1]. Refeeding complications can include the development the Refeeding Syndrome (RFS), and other metabolic and micronutrient changes. Shifts in electrolytes and fluid in response to increased caloric intake are implicated in the development of these complications [2].

Abnormal levels of phosphate, changes in macronutrient metabolism, as well as low extracellular levels of sodium, magnesium and potassium, are also used as markers of RFS [2]. Recently, Rio et al. (2013) [3] proposed more specific criteria for diagnosing RFS, encompassing electrolyte levels, presence of oedema and respiratory/cardiac dysfunction. In addition to RFS, thiamine deficiency (TD) is also listed as a concern when providing nutritional replenishment to malnourished patients, as severe deficiency can lead to Wernicke’s encephalopathy (WE) or Wernicke Korsakoff’s syndrome (WKS) [2].

In 2006, the National Institute for Health and Care Excellence (NICE) developed clinical guidelines to identify patients at risk of refeeding complications, including patients with a low body mass index (BMI) < 18.5 kg/m^2^, unintentional weight loss > 10% body weight in the last 3–6 months, and a recent history of little of no nutritional intake [4]. The updated NICE guidelines recommend supplementing with 200–300 mg oral thiamine daily prior to commencing feeding and during the first 10 days of feeding in people identified at risk of developing refeeding problems [4]. However, the paucity of scientific evidence underpinning these and other recommendations has been a key criticism of the NICE Clinical Guidelines, in the management of patients with eating disorders [5, 6]. There is also a growing body of evidence that challenges the ‘start low, go slow’ approach to renourishing patients with a restrictive eating disorder, as it has been shown to lead to the development of ‘underfeeding syndrome’, whereby malnourished patients are provided with insufficient energy intakes to meet basal metabolic needs and they continue to lose weight [7–12].

Routine supplementation of thiamine in patients with AN prior to initiation of nutritional rehabilitation is an example of a clinical guideline based on expert opinion rather than evidence-based recommendations [2, 4, 13–15]. This practice is based on foundation knowledge of the role of thiamine as an essential cofactor in carbohydrate metabolism, an essential pathway during refeeding [2], and previous research demonstrating thiamine supplementation prior to nutritional rehabilitation may prevent TD and its associated neuropsychiatric conditions WE and WKS [16, 17]. A study of 37 patients with AN reported a 38% prevalence of TD [18], and a systematic review by Oudman E et al. (2018) identified 12 cases of TD causing WE in patients with AN ranging from BMI 12.2 kg/m^2^ to 17.4 kg/m^2^, including some patients with a history of alcohol and substance abuse [19].

The aim of this study is to investigate whether adolescents hospitalised with a restrictive eating disorder and commenced on a high caloric refeeding regimen, present with or develop thiamine deficiency.

## Methods

### Study design

A retrospective audit of 119 consecutive admissions between July 2016–December 2017 was conducted. Inclusion criteria were patients diagnosed with a restrictive eating disorder requiring nutritional rehabilitation and at least one thiamine level measured during the admission. Patients were excluded from the study if they did not have a blood thiamine level assessed during admission. Those with repeat admissions were included in the analysis if the inclusion criteria was met for subsequent admissions.

Retrospective data from paper-based and electronic medical records were collected for each admission. This included patient demographics, diagnosis, weekly weight, body mass index (BMI), weekly caloric intake, oral electrolyte supplementation received, length of hospital stay, history of purging and laxative use and any clinical signs and symptoms of refeeding syndrome and thiamine deficiency, including delirium and peripheral oedema. Baseline and weekly blood thiamine levels as well as supplement prescription were also reviewed. This study received ethical approval from the Western Sydney Local Health District Human Research and Ethics committee.

### Ward program

**Fig. 1:**
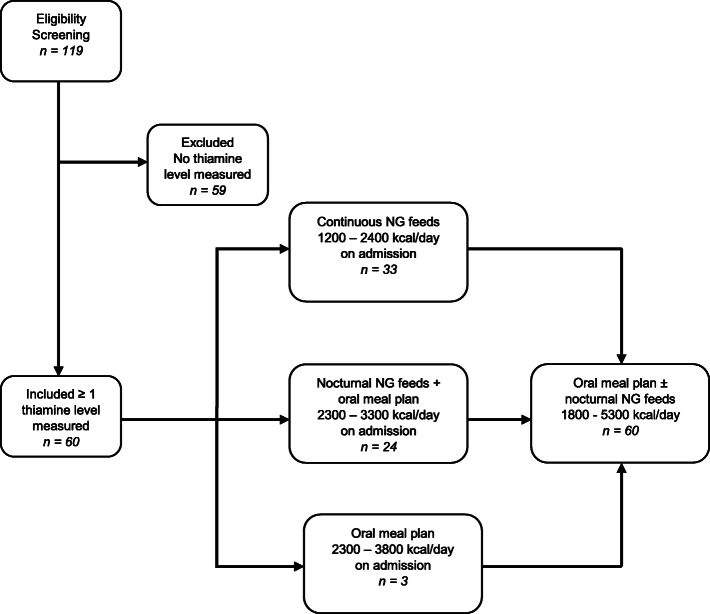
Nutrition rehabilitation program provided to patients on admission and the progression to oral dietary intake

On admission, patients were prescribed a standardised nutritional regimen consisting of either (i) continuous nasogastric feeds with oral intake limited to sips of water, (ii) nocturnal nasogastric feeds with an oral meal plan during the day, or (iii) oral meal plan only (Fig. 1).

Patients who were considered medically unstable (presenting with bradycardia, heart rate ≤ 50 bpm, QTc interval on ECG > 440 ms, or significantly hypotensive) were typically commenced on 1 kcal/mL formula of continuous nasogastric (NG) tube feeding to provide 2400 kcal/day at a rate of 100 mL/hour, or a 1.5 kcal formula at a rate of 35 mL/hr. for 12 h and then increased to 70 ml/hr. (providing 1890 kcal on Day 1). All oral intake other than water was restricted during the first 24 h.

Patients with an eating disorder who were assessed as medically stable were commenced on an oral meal plan providing 1800 to 3800 kcal with or without supplementary nocturnal NG feeds (1 kcal/mL formula at 100 mL/hour for 10 h providing 1000 kcal). If a meal was not consumed to completion, patients were provided with additional nutritional supplements to assure prescribed caloric intake.

A patient’s caloric intake was increased throughout hospitalisation by increasing meal plans (1800 kcal, 2300 kcal, 2800 kcal, 3300 kcal and 3800 kcal), providing nutritional supplements (300 kcal or 400 kcal), or altering the volume or concentration of enteral feeds (1.0, 1.5, 2.0 kcal/mL formula). The total caloric intake was reviewed at least 3 times/week by the treating medical team, where decisions were made in regards to modifying the total caloric intake.

Typically, patients received prophylactic phosphate supplementation and a daily multivitamin with the commencement of the nutritional regimen. The daily multivitamin provided 10 mg thiamine. The thiamine content in oral meal plans was 2.30–3.62 mg/day, and the thiamine content of Enteral Formula ranged from 1.6–3.2 mg/L. Serum electrolyte levels were monitored 6 h post nutritional intervention commencement and continued regularly until discharge. Biochemical analyses were based on the hospital reference ranges for thiamine 67–200 nmol/L. To measure thiamine levels, a commercial kit Chromsystems™ with combined Vitamin B1/B6 Analysis UHPLC/52952 was used, with an intra-assay coefficient of variation ≤3.8% and inter-assay coefficient of variation ≤6% [20].

### Statistical analysis

Descriptive analysis included patient demographics and clinical data. Number (*n*), range, mean, standard deviation, and percentages are reported. Variables recorded included caloric prescription on admission, admission weight, weight change, length of hospitalisation. Percentage median BMI (%mBMI), a calculation of the 50th percentile BMI for age and gender, was used as an indicator of malnutrition due to this being a routinely used measure in adolescent and young adult patients [21, 22].

Data were entered into SPSS for Windows Version 21, IBM Corporation and S-Plus for analysis. A linear mixed effects model conducted on thiamine trends during nutrition rehabilitation was used to analyse the data. Blood thiamine levels were assessed through data stacking. The random effect was plotted as week per individual admission. The fixed effect was weekly blood thiamine level and the value and standard error were assessed. Statistical significance was set at *p* value < 0.05.

## Results

During the 18 month study period, 119 consecutive admissions were identified and 60 admissions (50.4%) met the eligibility criteria for analysis (52 patients with 1 admission, 4 patients with 2 admissions). The patient cohort included 37 admissions diagnosed with DSM-5 criteria [23] for anorexia nervosa (restricting type), 14 admissions with anorexia nervosa (binge eating/purging type), and 6 admissions with Avoidant/Restrictive Food Intake Disorder. Three admissions were diagnosed with an Atypical anorexia nervosa. All patients received the daily multivitamin supplement.

Fifty-nine cases were excluded as thiamine level was not tested during their admission. Of the 60 patients included in the study, 88% (*n* = 53) were female. On admission, mean age was 17.2 years (SD 1.2), percentage mBMI was 80.4% (SD 8.9). Patients were malnourished (11.7% severe, 31.7% moderate, 56.7% mild) [22]. Additional patient characteristics are presented in Table 1.

**Table 1:** Characteristics of adolescent patients hospitalised with a restrictive eating disorder (60 admissions, 56 patients)

Variable	Mean (SD)	Median (LQ-UQ)
Age (years)	17.2 (1.2)	17.2 (16.4–18.1)
Admission Weight (kg)	45.8 (7.3)	45.8 (41.3–51.9)
Discharge weight (kg)	53.4 (6.6)	53.6 (48.9–57.0)
Admission BMI (kg/m2)	16.8 (1.8)	17.1 (15.8–18.3)
Discharge BMI (kg/m2)	19.5 (1.2)	19.7 (18.7–20.2)
Admission %mBMI	80.4 (8.9)	81.3 (75.2–87.2)
Discharge %mBMI	93.2 (6.3)	94.3 (89.4–97.0)
Change in %mBMI	12.8 (6.6)	12.0 (7.4–16.6)
Energy intake on admission (kcal)	2482.0 (413.2)	2400.0 (2400.0–2800.0)
Energy intake on discharge (kcal)	3753.3 (617.7)	3800.0 (3400.0–3800.0)
Total weight gain (kg)	6.9 (4.4)	6.1 (4.2–8.9)
Average weight gain per week (kg)	2.0 (0.8)	2.0 (1.5–2.4)
Average LOS (weeks)	3.6 (2.0)	3.2 (2.2–4.6)

*BMI* Body mass index, *%mBMI* Percentage median body mass index, *LOS* Length of hospital stay.

**Fig. 2:**
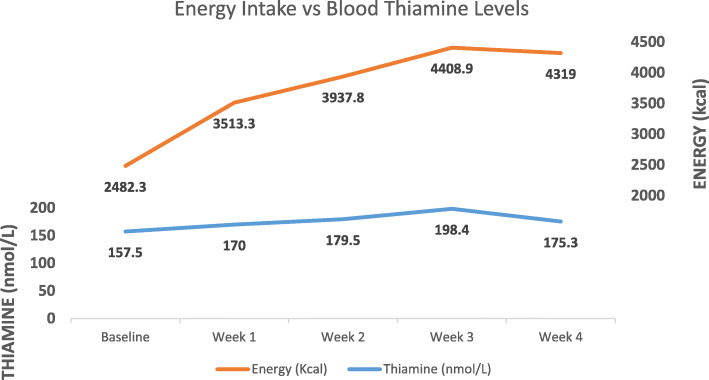
Changes in energy intake and blood thiamine levels *n* = 60

Initial nasogastric (NG) tube feeding and/or oral intake was introduced and an average of 2482.0 kcal (SD 413.2) was prescribed, and was sequentially increased during the admission (Fig. 2).

**Fig. 3:**
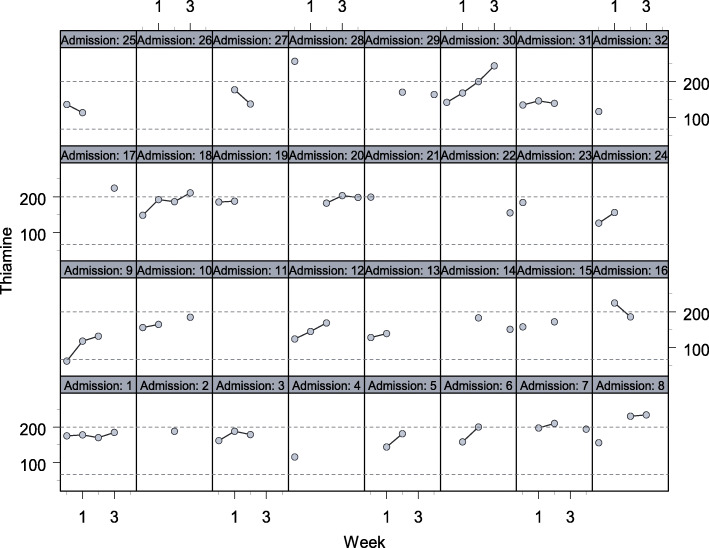
Linear mixed effects model stacking patient thiamine outcome per week of Patients 1–32

The mean thiamine level on admission was 157.5 nmol/L (SD 36.0). During hospitalisation and nutrition intervention, median thiamine levels increased by 9.2 nmol per week (SD 2.4, IQR 4.4–14, *p* value < 0.001), based on the linear mixed effects model (Fig. 3). There was only one occasion, representing 1.7% of the study sample, when a patient had blood thiamine level recorded below the normal reference range at 62 nmol/L, on admission, prior to nutrition intervention. The remaining thiamine levels on admission were all within the normal reference range of 67 – 200 nmol/L. Over the duration of admission, 15 out of 60 patients (25%) were observed to have a blood thiamine level greater than the upper limit of the normal reference range (200 nmol/L).

## Discussion

Adolescents hospitalised with a restrictive eating disorder and commenced on a high caloric prescription did not require additional thiamine supplementation, beyond 10 mg contained within a daily multivitamin, to maintain normal levels of thiamine during nutritional rehabilitation. Blood thiamine levels increased weekly during their admission, most likely due to thiamine intake from a daily oral multivitamin, dietary food intake and enteral feeds.

Neither low thiamine levels nor many of the proposed predisposing factors for low thiamine, are typically present for adolescents with mild to moderate malnutrition presenting with restrictive eating disorders. Nevertheless, guidelines for the nutritional management of patients diagnosed with AN include the prophylactic supplementation of 200-300 mg thiamine as a preventative measure for the development of refeeding complications [13]. Restriction of thiamine rich foods, excessive excretion of magnesium, and ethanol toxicity are suggested as causes for low thiamine levels and the development of WE in patients with starvation and restrictive eating disorders [17, 24–28]. There appears to be uncertainty in the literature regarding the prevalence of thiamine deficiency observed in malnourished patients, relating to two different methods of analysis, i.e. erythrocyte transketolase activity versus measurement of blood thiamine.

Low levels of erythrocyte transketolase activity have been reported in as many as 38% of patients diagnosed with AN [18]. In the present study only one patient presented with TD on admission, as measured by blood thiamine level. The higher prevalence of thiamine deficiency in AN reported by Winston AP (2000) [18] may be explained by the use of a different method of assessment in addition to investigating an older population with possibly a longer duration of illness. In their study of 37 patients, the authors reported no statistical relationship between erythrocyte transketolase activation and age, BMI, duration of restricted oral intake, frequency of vomiting, use of laxatives or alcohol consumption [18]. Reduced erythrocyte transketolase activity is likely a result of malnutrition rather than a refeeding complication, and does not necessarily infer reduced levels of thiamine.

No patients developed TD in this study. Above-RDI levels of thiamine were provided via oral meal plans, daily multivitamin supplementation and enteral feeds, as part of standard-care practices. The RDI for thiamine for patients admitted to the adolescent ward ranges from 1.1 mg/day – 1.2 mg/day, depending on gender and age [29], and the multivitamin alone provided patients with 10 mg of thiamine daily (approximately 10 times the RDI). Comparison of these findings to other studies is difficult because the amount of thiamine consumed from oral intake and enteral feeds is not being routinely reported [18, 19].

Recommendations for the management of TD in malnourished patients continues to be influenced by guidelines for alcohol dependent patients. Thiamine deficiency in alcohol dependent individuals may result from inadequate thiamine intake, however it may also be a complication of ethanol toxicity leading to reduced intestinal absorption and bioavailability of thiamine, as well as increased thiamine requirement needed for the metabolism of alcohol [16, 17]. Furthermore, magnesium depletion has shown effects of aggravating thiamine deficiency, whereby alcohol ingestion leads to increased excretion of magnesium and may expedite the presentation of WE symptoms [24]. Guidelines for preventing refeeding complications [2, 4, 30], including patients with AN [13], recommend 200–300 mg/day thiamine supplementation, which is similar to the lower end of the range recommended in guidelines for preventing and treating TD and WE in alcohol dependent individuals which range from 200 to 600 mg/day thiamine supplementation [17, 31, 32].

A limitation of the present study is the absence of thiamine measurements for 49.6% of the study population, and only one method of analysis was measured. The retrospective collection of data means the study was bound by what information was documented during the admission. The current study did not identify any clinical symptoms of WE such as mental confusion or delirium, however routine assessments for ataxia and ocular motility abnormalities such as nystagmus and opthalmoplegia, are not part of routine care and may have been missed. As part of routine care, if any cases of delirium had been detected during inpatient treatment, the patient would have been referred for further investigation including clinical signs of TD. Another limitation of the study is that patients had predominantly mild to moderate malnutrition. TD and refeeding complications are more likely to develop in those with severe malnutrition, so any recommendations from this study can only apply to mild and moderately malnourished patients.

Future studies would benefit from assessing prospectively the typical symptoms of WE, including ataxia, opthalmoplegia, nystagmus and confusion on admission and throughout nutritional rehabilitation. Furthermore, future studies may consider using multiple methods for assessing thiamine status including measurement of urinary thiamine excretion as well as MRI in suspected cases of WE.

There is scope for this study to be expanded beyond an investigation of thiamine status in adolescent patients with eating disorders to include all patients who present with malnutrition and are at risk of thiamine deficiency and refeeding complications on admission. A greater sample size using a prospective study design will increase the validity of the study. Furthermore, by assessing different ages, genders and contributing comorbidities, the generalisability of the study will be expanded and translation into practice will be more impactful.

## Conclusions

In conclusion, the results of the study suggest that providing high doses of thiamine through additional supplementation may be unnecessary in mild and moderately malnourished adolescents hospitalised with a restrictive eating disorder. Considering there were no cases of patients developing TD during the admission, it would suggest that patients received adequate thiamine levels through the nutritional rehabilitation program in the current study, through oral diet, enteral feeds and a daily multivitamin.

## Data Availability

The datasets used and/or analysed during the current study are available from the corresponding author on reasonable request.

## Abbreviations

AN: Anorexia nervosa
RFS: Refeeding syndrome
TD: Thiamine deficiency
WE: Wernicke’s encephalopathy
WKS: Wernicke-Korsakoff syndrome
BMI: Body mass index
% mBMI: Percentage median body mass index
NG: Nasogastric
